# Disease-modifying effects of ranibizumab for central retinal vein occlusion

**DOI:** 10.1007/s00417-021-05224-x

**Published:** 2021-10-06

**Authors:** Jason M. Huang, Rahul N. Khurana, Avanti Ghanekar, Pin-wen Wang, Bann-Mo Day, Barbara A. Blodi, Amitha Domalpally, Carlos Quezada-Ruiz, Michael S. Ip

**Affiliations:** 1 University of California Los Angeles Stein Eye Institute, Los Angeles, CA USA; 2 Northern California Retina Vitreous Associates, Mountain View, CA USA; 3 Department of Ophthalmology, University of California San Francisco, San Francisco, CA USA; 4 Genentech Inc, South San Francisco, CA USA; 5 Department of Ophthalmology and Visual Sciences, School of Medicine and Public Health, University of Wisconsin, Madison, WI USA; 6 Retina Y Vitreo, Clinica de Ojos de La Garza Viejo, San Pedro Garza Garcia, Mexico; 7 Doheny Eye Institute, 800 South Fairmont Avenue, Pasadena, CA 91105 USA

**Keywords:** Retina, Neovascularization, Drugs, Treatment medical

## Abstract

**Purpose:**

To identify anatomic endpoints altered by intravitreal ranibizumab in central retinal vein occlusion (CRVO) to determine any potential underlying disease modification that occurs with anti-vascular endothelial growth factor (anti-VEGF) therapy beyond best-corrected visual acuity and central optical coherence tomography outcomes.

**Methods:**

A post hoc analysis of a double-masked, multicenter, randomized clinical trial was performed. A total of 392 patients with macular edema after CRVO were randomized 1:1:1 to receive monthly intraocular injections of 0.3 or 0.5 mg of ranibizumab or sham injections. Central reading center-read data were reviewed to explore potential anatomic endpoints altered by therapy.

**Results:**

At 6 months, there was a reduction in the ranibizumab groups compared with sham groups with respect to total area of retinal hemorrhage (median change from baseline in disc areas: − 1.17 [sham], − 2.37 [ranibizumab 0.3 mg], − 1.64 [ranibizumab 0.5 mg]), development of disc neovascularization (prevalence: 3% [sham], 0% [ranibizumab 0.3 mg], 0% [ranibizumab 0.5 mg]), and presence of papillary swelling (prevalence: 22.9% [sham], 8.0% [ranibizumab 0.3 mg], 8.3% [ranibizumab 0.5 mg], *p* < 0.01). There was no difference between groups in collateral vessel formation. Analysis of vitreous and preretinal hemorrhage could not be performed due to low frequency of events in both treated and sham groups.

**Conclusions:**

Ranibizumab for CRVO resulted in beneficial disease-modifying effects through a reduction in retinal hemorrhage, neovascularization, and papillary swelling. These findings may form the basis for future work in the development of a treatment response or severity scale for eyes with CRVO.


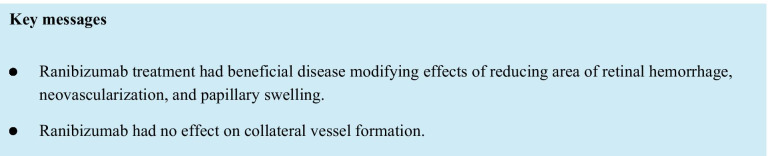


## Introduction

Central retinal vein occlusion (CRVO) is a retinal vascular condition that affects an estimated 16 million people worldwide [1]. The pathophysiology of CRVO involves ischemia through occlusion of the lumen of the central retinal vein, which can lead to increased levels of vascular endothelial growth factor (VEGF). Elevated levels of VEGF can cause macular edema and neovascularization, subsequently leading to vision loss [2, 3]. Anti-vascular endothelial growth factor (anti-VEGF) agents such as bevacizumab, ranibizumab, and aflibercept have been shown to reduce CRVO-related ocular morbidity via improvement in visual acuity and reduction in macular edema [4–7].

The phase III Ranibizumab for the Treatment of Macular Edema after Central Retinal Vein Occlusion Study: Evaluation of Efficacy and Safety (CRUISE) trial (NCT 00,485,836) was a double-masked, multicenter, randomized study that showed the benefit of ranibizumab in eyes with CRVO. Patients treated with ranibizumab were found to have improved visual acuity and macular edema at the 6-month endpoint [4]. Further subanalysis of this dataset has also revealed improved macular non-perfusion in patients treated with ranibizumab [8]. Due to the significant improvement in patients treated with ranibizumab compared to sham, anti-VEGF therapy is now the standard of care for CRVO, and the CRUISE trial was hence one of the last sham-controlled CRVO trials.

Treatment with anti-VEGF therapy has been found to lead to disease modification in diabetic retinopathy [9, 10]. For example, the RIDE/RISE study (NCT00473382/00,473,330) showed that with ranibizumab monthly treatment, in addition to beneficial changes in visual acuity and macular edema, diabetic retinopathy severity could be improved over time [11–13]. Given the disease-modifying effects present in diabetic retinopathy, we hypothesized that anti-VEGF therapy could also produce underlying disease modification in CRVO as well. The BRAVO and CRUISE studies in BRVO and CRVO, respectively, are among the last recent examples of sham-controlled randomized clinical studies where natural history can be compared to the effects of anti-VEGF therapy. The present analysis reviewed available CRUISE data (read by the University of Wisconsin Fundus Photograph Reading Center) to identify additional anatomic and clinical endpoints that could signify beneficial disease modification following anti-VEGF therapy.

## Materials and methods

CRUISE (clinicaltrials.gov identifier NCT00485836) was a phase III, multicenter study that showed the efficacy and safety of ranibizumab intravitreal injections compared with sham injections in patients with macular edema secondary to CRVO. In that trial, 392 participants were randomized to one of three treatment arms involving 6 monthly injections of (1) sham injection, (2) ranibizumab injection 0.3 mg, or (3) ranibizumab 0.5 mg. Compared with the sham arm, both ranibizumab treatment arms were found to have improved best-corrected visual acuity and central foveal thickness at month 6 [4].

In the present study, analysis of anatomic variables not previously explored in the CRUISE study was performed to better understand the disease modification that occurs with ranibizumab. Institutional Review Board approval was obtained for the original CRUISE study, and this research adhered to the tenets of the Declaration of Helsinki. Color fundus photographs, time domain optical coherence tomography images, and fluorescein angiogram images were collected for baseline, 3-month, and 6-month visits. Color fundus photographs were obtained using a 30° fundus camera and were performed as an array of three photographic fields. The images were assessed for type of vein occlusion, area of retinal hemorrhage, neovascularization of the disc, papillary swelling, retinal collateral vessels, vitreous hemorrhage, and preretinal hemorrhage. Fluorescein angiography was assessed for capillary non-perfusion, retinal leakage area, and neovascularization. Area of retinal hemorrhage was assessed within the ETDRS grid placed on the field 2 image centered on the macula. The percentage of hemorrhage in each subfield of the ETDRS grid was estimated. Subsequently, an algorithm was applied using known area values of each subfield to convert the percentage estimates into absolute area of retinal hemorrhage in disc areas. Papillary swelling was assessed in comparison to reference photographs. All images were analyzed by the University of Wisconsin Fundus Photograph Reading Center.

This post hoc analysis examined total area of retinal hemorrhage (in disc areas [DA]) as a continuous endpoint. The following binary (absent vs. definite) categorical endpoints were examined: neovascularization of the disc, papillary swelling, collateral vessels within the retina, vitreous hemorrhage, and preretinal hemorrhage. For continuous variables, descriptive summaries (mean and standard deviation, or median and interquartile range) for change from baseline over time were reported for each treatment group. For categorical variables, count and percentages were reported over time, and Pearson chi-square tests or Fisher’s exact test was used to compare between the treatment groups at each time point. Data were excluded if there were missing images, ungradable poor-quality images, incomplete assessments, or missing visits. Statistical analyses were performed using the SAS software version 9.4 (SAS Institute Inc., Cary, NC).

## Results

**Fig. 1:**
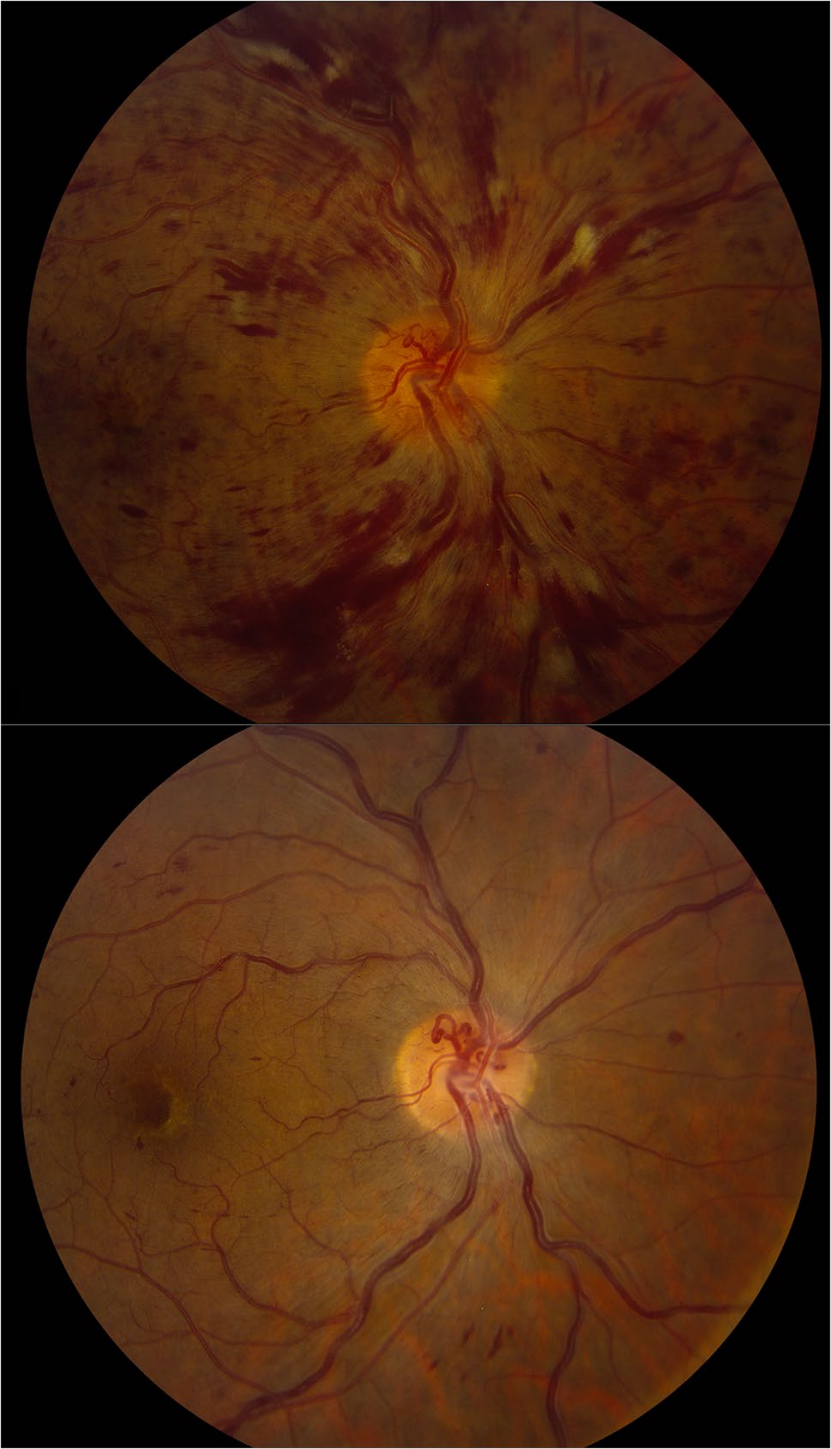
Baseline (top) and follow-up (bottom) photos of an eye receiving ranibizumab treatment. Treatment improved retinal hemorrhage area and papillary swelling but had no effect on collaterals on the disc

The 392 participants from the CRUISE trial were included in the present study. The results are summarized in Table 1. Representative images of the effects of ranibizumab treatment are also displayed in Fig. 1.

- Retinal hemorrhage: Out of 391 study eyes included in this analysis, 130 were in the sham group, 131 were in the ranibizumab 0.3 mg group, and 130 were in the ranibizumab 0.5 mg group at baseline. By month 3, total area of retinal hemorrhage changed by a median of − 0.42 (interquartile range [IQR] − 2.96 to 0.21) disc areas (DA) in the sham group, − 1.78 (IQR − 3.88 to − 0.37) DA in the ranibizumab 0.3 mg group, and − 1.45 (IQR − 3.97 to − 0.41) DA in the ranibizumab 0.5 mg group. At month 6, retinal hemorrhage area changed by a median of − 1.17 (IQR − 4.86 to − 0.06) DA in the sham group compared to − 2.37 (IQR − 5.11 to − 0.41) and − 1.64 (IQR − 4.35 to − 0.53) DA in the ranibizumab 0.3 and 0.5 mg groups, respectively.
- Neovascularization of the disc: Out of 391 total eyes, none had neovascularization of the disc at baseline. At month 3, 2 out of 104 patients (1.9%) developed new vessels at the disc in the sham group; 0 out of 118 patients (0%) and 0 out of 112 patients (0%) developed any new vessels at the disc in the ranibizumab 0.3 mg and 0.5 mg group, respectively. At month 6, 3 out of 99 patients (3%) in the sham group developed new vessels at the disc. A total of 0 out of 119 patients (0%) and 0 out of 106 patients (0%) developed any new vessels at the disc in the ranibizumab 0.3 mg and 0.5 mg group, respectively.
- Papillary swelling: Analysis of papillary swelling included 265 eyes (83 sham, 89 ranibizumab 0.3 mg, 93 ranibizumab 0.5 mg) at baseline. The percentage of patients with definite papillary swelling at baseline was 38.6% (*n* = 32/83), 46.1% (41/89), and 41.9% (39/93) in the sham, ranibizumab 0.3 mg, and ranibizumab 0.5 mg groups, respectively. At month 3, the proportion of patients with papillary swelling was 29.3% (24/82), 17.2% (17/99), and 19.2% (19/99), respectively (sham vs. ranibizumab 0.3 mg *p* = 0.05, sham vs. ranibizumab 0.5 mg *p* = 0.11). At month 6, the proportion was 22.9% (19/83) in the sham group compared to 8.0% (8/100) and 8.3% (8/96) in the ranibizumab 0.3 mg and 0.5 mg groups, respectively (sham vs. ranibizumab 0.3 mg *p* < 0.01, sham vs. ranibizumab 0.5 mg *p* < 0.01).
- Collateral vessels: The occurrence of collateral vessels within the retina was analyzed in 366 patients (119 sham, 125 ranibizumab 0.3 mg, 122 ranibizumab 0.5 mg). Collateral vessels were observed at baseline in 7.6% (9/119), 10.4% (13/125), and 6.6% (8/122) of eyes in the sham, ranibizumab 0.3 mg, and ranibizumab 0.5 mg groups, respectively. These numbers remained stable at month 3 at 6.0% (5/84) for sham, 11.5% (12/104) for ranibizumab 0.3 mg, and 11.5% (11/96) for ranibizumab 0.5 mg (sham vs. ranibizumab 0.3 mg *p* = 0.18, sham vs. ranibizumab 0.5 mg *p* = 0.20). The numbers were stable again at month 6 with 11.5% (11/96) for sham, 6.5% (7/107) for ranibizumab 0.3 mg, and 7.3% (7/96) for ranibizumab 0.5 mg (sham vs. ranibizumab 0.3 mg *p* = 0.22, sham vs. ranibizumab 0.5 mg *p* = 0.32).
- Vitreous and preretinal hemorrhage: A total of 330 subjects were analyzed at baseline for vitreous or preretinal hemorrhage (130 sham, 131 ranibizumab 0.3 mg, 130 ranibizumab 0.5 mg). At baseline, there were 0 (0%), 2 (1.5%), and 3 (2.3%) occurrences of preretinal hemorrhage in sham, ranibizumab 0.3 mg, and ranibizumab 0.5 mg, respectively. There was one (1.0%) occurrence of preretinal hemorrhage in the sham group, and none in the ranibizumab groups at 6 months. There were no occurrences of vitreous hemorrhage in any group at baseline or at 6 months. Due to the low number of events, the effect of ranibizumab on vitreous and preretinal hemorrhage could not be determined.
- Fluorescein angiogram leakage: Total area of fluorescein leakage was measured in 387 patients (128 sham, 130 ranibizumab 0.3 mg, 129 ranibizumab 0.5 mg). At baseline, the mean area of leakage was 12.19 (standard deviation [SD] 4.60), 11.92 (SD 4.85), and 11.91 (SD 4.67) DA for sham, ranibizumab 0.3 mg, and ranibizumab 0.5 mg, respectively. At month 3, the mean area of leakage was 11.37 (SD 4.75), 5.84 (SD 5.54), and 4.83 (SD 5.30) DA; at month 6, the mean area was 10.03 (SD 5.15), 3.61 (SD 4.58), and 3.07 (SD 4.69) DA for sham, ranibizumab 0.3 mg, and ranibizumab 0.5 mg, respectively.

**Table 1:** Summary of results

	Sham	Ranibizumab 0.3 mg	Ranibizumab 0.5 mg
Change in retinal hemorrhage area from baseline (median, disc areas)			
• 3 months	− 0.42 (IQR − 2.96 to 0.21)	− 1.78 (IQR − 3.88 to − 0.37)	− 1.45 (IQR − 3.97 to − 0.41)
• 6 months	− 1.17 (IQR -4.86 to -0.06)	− 2.37 (IQR − 5.11 to − 0.41)	-1.64 (IQR -4.35 to − 0.53)
Neovascularization of the disc			
• Baseline	0/130 (0%)	0/131 (0%)	0/130 (0%)
• 3 months	2/104 (1.9%)	0/118 (0%)	0/112 (0%)
• 6 months	3/99 (3.0%)	0/119 (0%)	0/106 (0%)
Papillary swelling			
• Baseline	32/83 (38.6%)	41/89 (46.1%)	39/93 (41.9%)
• 3 months	24/82 (29.3%)	17/99 (17.2%) (*p* = 0.05)	19/99 (19.2%) (*p* = 0.11)
• 6 months	19/83 (22.9%)	8/100 (8.0%) (*p* < 0.01)	8/96 (8.3%) (*p* < 0.01)
Collateral vessels			
• Baseline	9/119 (7.6%)	13/125 (10.4%)	8/122 (6.6%)
• 3 months	5/84 (6.0%)	12/104 (11.5%) (*p* = 0.18)	11/96 (11.5%) (*p* = 0.20)
• 6 months	11/96 (11.5%)	7/107 (6.5%) (*p* = 0.22)	7/96 (7.3%) (*p* = 0.32)

## Discussion

Ranibizumab treatment resulted in rapid and greater reduction of retinal hemorrhage area and papillary swelling compared with sham, and treatment reduced the rate of neovascularization of the disc. Exploratory analysis also found that ranibizumab treatment resulted in decrease in fluorescein angiogram leakage area. These differences were seen as early as 3 months. Corresponding to these findings of disease modification occurring rapidly after anti-VEGF treatment, the CRUISE trial showed that changes in visual acuity and optical coherence tomography central subfield thickness occurred soon (as early as 7 days) after initiation of anti-VEGF therapy [4]. Previous research has also shown that median time to 10-letter gain in the CRUISE study was 2.3 months and 2.0 months in the 0.3 mg and 0.5 mg ranibizumab groups, respectively, compared to 8.2 months in the sham group that was crossed over to 0.5 mg ranibizumab after 6 months without treatment. Additionally, 78.9% and 81.3% of eyes in the 0.3 mg and 0.5 mg ranibizumab groups achieved ≥ 10-letter gain compared to just 63.0% of eyes in the crossover sham/0.5 mg ranibizumab group [14]. In a prior analysis of the same dataset analyzed herein, by Campochiaro et al., the additional benefit of a protective effect of ranibizumab on the development retinal non-perfusion was noted [8]. These observations suggest the importance of early treatment of macular edema from CRVO, as anti-VEGF therapy seems to provide a rapid and beneficial disease-modifying effect that may contribute to improved vision.

Previous studies evaluating intravitreal ranibizumab and aflibercept for diabetic macular edema show that these anti-VEGF therapies can lead to improvement in Diabetic Retinopathy Severity Score (DRSS) [11, 12, 15–17]. While different diseases, the present study shows that ranibizumab also appears to have a similar disease-modifying effect for retinal vein occlusion. Retinal vein occlusion and diabetic retinopathy share a common pathophysiologic mechanism of ischemia leading to VEGF upregulation [18, 19]. VEGF has been shown to induce changes in tight junction proteins occludin and zonula occluden 1 [20]. VEGF has also been shown to cause vascular leakage through pre-existing vessels and growth of anatomically abnormal vessels [3, 21, 22]. Blocking VEGF in patients with diabetic retinopathy and vein occlusion reduces vascular permeability, which in turn may help reduce retinal hemorrhage [23, 24]. Therefore, ranibizumab and other anti-VEGF agents may allow for a more rapid resolution to normal retinal circulation than if left to natural history alone.

Post hoc analysis of phase III RISE and RIDE trials showed that DRSS improvement was correlated with better mean improvement in best-corrected visual acuity, contrast sensitivity, and resolution of macular edema [25]. Similar to DRSS for diabetes, the quantity of retinal hemorrhages, amount of neovascularization of the disc, and level of papillary swelling could be used to create a severity scale for CRVO, which may be helpful for classification and prognostication. This can be particularly important for vein occlusion because although the occlusive event is acute, the HORIZON and RETAIN studies have shown that many eyes require long-term management [26, 27]. Additionally, the presentation and level of ocular morbidity are variable, and it can be difficult to predict which patients are more likely to experience more devastating vision loss or more recurrent macular edema. Changes in retinal ischemic index, for example, have been correlated with recalcitrant macular edema in retinal vein occlusion [28]. Correlating other anatomic endpoints and recurrent macular edema could lead to better prognostication and more individualized treatment protocols for the management of retinal vein occlusion.

Our findings here are consistent with another publication on the CRUISE results, which examined change in the number of retinal hemorrhages as a secondary outcome [29]. In the prior publication of the CRUISE trial, clinical examiners were asked to categorically determine whether patients had zero hemorrhages or > 10 hemorrhages based on the indirect exam. In contrast, this analysis calculated the total geographic area of retinal hemorrhages within the macula in color fundus photos using trained graders at a reading center with standardized grading protocols, likely increasing accuracy of the grading. Additionally, assessing area of retinal hemorrhage rather than counting the number of hemorrhages is a better reflection of how much of the fundus is affected by this feature of retinal vein occlusion. This provides a more accurate quantitative measurement that may be useful for the development of a future severity scale.

Although we found a decreased rate of neovascularization of the disc, Brown et al. did not find a decrease in the rate of rubeosis in patients treated with ranibizumab for CRVO [30]. In that study, neovascular complications were merely delayed. However, ischemic (≥ 10 disc areas of non-perfusion) CRVOs were included in the Brown et al. study, whereas the present analysis included predominately non-ischemic (< 10 disc areas of non-perfusion) CRVOs. Additionally, follow-up time in that study was 36 months compared to 6 months in the original phase III CRUISE trial, and thus it is possible that neovascularization may have resulted at a later time after the completion of the original CRUISE study, when patients were no longer being treated per protocol after exiting the CRUISE clinical study. It would be interesting to review 12-month CRUISE study results [29] or longer-term follow-up from the RETAIN study [27] to examine if the rate of neovascularization increases despite long-term therapy with anti-VEGF.

We did not find a reduction in collateral vessels with ranibizumab treatment. This is consistent with prior work that showed that neither ranibizumab nor triamcinolone had an effect on the development of venous collaterals. Furthermore, these studies revealed that presence of collateral vessels was not associated with visual acuity [31, 32]. Collateral vessels may represent permanent dilation and changes to native retinal vessels, which may explain the lack of influence that intravitreal injections have on collateral vessel formation.

The major strengths of this report are that data were obtained from a large, double-masked, multicenter, randomized trial, and that images were obtained at regular intervals and evaluated by an independent reading center using graders masked to the treatment assignment. Another strength of the study is that the control arm did not receive treatment and thus reflects the natural history of the disease. Limitations of this study include the post hoc nature of this analysis. Additionally, images in the CRUISE study were obtained with a 30° fundus camera, so wide-field retinal findings could not be assessed. Follow-up time comparing sham to ranibizumab was limited to 6 months, and it is not known whether the effects on anatomic endpoints persist beyond 6 months or if they recur following cessation or reduction in treatment frequency. Additionally, due to the limited number of preretinal and vitreous hemorrhage events regardless of sham or treatment arm, analysis of these endpoints could not be performed.

In summary, we found that ranibizumab provides beneficial disease modification in eyes with CRVO through 6 months of treatment in addition to the well-known effects of ranibizumab on visual acuity and macular edema. This study provides the initial framework for future research on the clinical importance of these anatomic endpoints. Ultimately, the evaluation, classification, and quantification of these anatomic endpoints could potentially be used for determining prognosis and a severity scale (much like that used for diabetic retinopathy) for eyes with CRVO.

## Data Availability

Data are available upon reasonable request.
